# Factors associated with psychological insulin resistance among patients with type 2 diabetes in China

**DOI:** 10.3389/fendo.2024.1368132

**Published:** 2024-07-05

**Authors:** Shilong Zhang, Xindan Zhang, Haipeng Wang, Wenyu Fan, Xingli Ma, Suhang Song, Beibei Zhang

**Affiliations:** ^1^ Centre for Health Management and Policy Research, School of Public Health, Cheeloo College of Medicine, Shandong University, Jinan, Shandong, China; ^2^ NHC Key Lab of Health Economics and Policy Research, Shandong University, Jinan, Shandong, China; ^3^ Department of Health Policy and Management, College of Public Health, University of Georgia, Athens, GA, United States; ^4^ Department of Clinical Laboratory, Shandong Mental Health Center, Jinan, Shandong, China

**Keywords:** psychological insulin resistance, My Opinion on Insulin, type 2 diabetes, China, insulin therapy

## Abstract

**Objective:**

The aim of this study was to understand the psychological insulin resistance status among Chinese patients with type 2 diabetes and investigate its associated factors in these patients.

**Methods:**

A multi-stage stratified random sampling was performed to randomly select patients with type 2 diabetes from the eastern, central, and western regions in Shandong Province, China, and 660 valid questionnaires were collected. Psychological insulin resistance was assessed by the scale of My Opinion on Insulin (MOI). Factors associated with psychological insulin resistance were examined in a binary logistic model.

**Results:**

Four-fifths of the patients with type 2 diabetes (82.1%) had psychological insulin resistance. Being female (OR = 1.770, 95% CI: 1.063–2.950, *p* < 0.05), having a monthly income of greater than 4,000 Renminbi (approximately $1,540) (OR = 0.444, 95% CI: 0.216–0.915, *p* < 0.05), living with type 2 diabetes for 11 years or more (OR = 0.387, 95% CI: 0.238–0.630, *p* < 0.05), self-rated poor health (OR = 1.706, 95% CI: 1.092–2.664, *p* < 0.05), and moderate discrimination against type 2 diabetes (OR = 1.924, 95% CI: 1.166–3.175, *p* < 0.05) were associated with psychological insulin resistance.

**Conclusions:**

The prevalence of psychological insulin resistance among Chinese patients with type 2 diabetes is relatively high. Approaches are needed to address the issue of psychological insulin resistance of type 2 diabetes.

## 1 Introduction

Type 2 diabetes (T2D) is a global chronic disease that requires continuous medical care and support programs (1). According to the latest International Diabetes Federation (IDF), the global prevalence of T2D in adults was 10.5% (536.6 million people) in 2021, and the number of people living with diabetes is estimated to be 12.2% (783.2 million) worldwide by 2045 (2). The latest research suggests 11% of adults living with diabetes in China, and the prevalence of T2D in Chinese individuals aged ≥65 years is 18.8% (3, 4). Various complex reasons contribute to the increased prevalence of T2D, such as aging and an unhealthy lifestyle (5–7). With the growing rate of aging population and poor management of T2D, the prevalence continued to increase in China (3, 4). T2D imposes a significant financial burden on individuals, families, healthcare systems, and countries (1). A study suggested that approximately $110 billion was spent on the treatment and management of patients with diabetes in China in 2015, accounting for 12% of the total medical expenditure (8).

To prevent or delay the occurrence of complications, it is necessary to monitor and control blood glucose (9). However, a considerable number of patients with T2D have inadequate glycemic control (10). Research has found that intensive glucose control is associated with a significant reduction in the risk of myocardial infarction and microvascular disease (11). For patients with poor glycemic control, delayed treatment can increase the risk of myocardial infarction (12). Insulin continues to be underutilized although multiple studies reported that insulin therapy was associated with improved glycemic control (13). It is crucial to address the issue of insufficient insulin utilization.

Current evidence showed that 18%–28.2% of patients were unwilling to start using insulin, 38%–44.8% of patients believed that insulin interfered with and restricted their daily lives, and 38%–78% of patients believed that insulin was the last way to treat diabetes (14, 15). Insulin treatment hesitation is affected by various factors, such as healthcare system barriers, healthcare professional barriers, and patient barriers (16, 17). Both healthcare professionals and patients may experience delays in insulin treatment, which poses a challenge to the control of patient blood sugar and subsequent treatment (18). Psychological insulin resistance (PIR) refers to psychological barriers to the initiation and persistence of insulin therapy (19). Paying attention to PIR may assist in addressing the hesitation of patients to undergo insulin therapy.

PIR is a complex balancing process, and the motivation for PIR formation varies between primary healthcare professionals and patients. Fears of insulin injection pain, concerns about insulin affecting daily life, costs of insulin treatment, problematic hypoglycemia, and misconceptions about insulin treatment are common reasons of the unwillingness to use insulin (14, 20–22). A survey of patients and doctors from multiple countries found that inadequate glycemic control is associated with non-adherence to insulin therapy, with 54.5% of doctors citing insulin injections as difficult at the time of prescription (23). A lack of experience in insulin initiation may hinder doctors from providing insulin treatment recommendations to patients (24, 25). A study found that primary care doctors are more likely to experience insulin treatment hesitation compared to specialist doctors (26). Primary care doctors and patients may be cautious about insulin initiation due to considerations of weight gain (27). The attitudes and concerns of doctors and patients toward insulin treatment may interact with each other. Meanwhile, the provider-to-patient relationship may also play an important role in patient insulin treatment (24).

The Diabetes Attitudes, Wishes, and Needs (DAWN) study can be used to assess patients’ attitude of insulin treatment. As an international cooperation project, it was initiated in 2001 and a revised version was launched again in 2013. There is relatively little research on the status and associated factors of PIR among patients with T2D at the national level, such as the status and associated factors of PIR among Chinese patients with T2D (28, 29). Considering the cultural differences, economic development levels, and healthcare service levels in different countries, patients’ perception or performance of PIR may vary. Therefore, this article aims to examine the levels and factors associated with PIR in Chinese patients with T2D, which could provide insight into glycemic control in patients with T2D.

## 2 Methods

### 2.1 Study design and setting

This study used the data collected from a cross-sectional survey conducted from November 2022 to March 2023 in Shandong Province, China. Shandong Province is located in eastern China and has a similar geographical and economic distribution to the country (30).

A multi-stage stratified random sampling method was used to recruit patients with T2D. First, three prefectures were selected based on their geographical location and socio-economic status in Shandong Province. Second, one urban district and one rural county were selected in each prefecture. Third, three streets or three townships were selected in each district and each county, respectively. Fourth, three villages were selected in each street and each township, respectively. Finally, a total of 27 villages were selected as sample areas. In each sampled village, 23–25 patients with T2D were randomly selected to conduct face-to-face interviews.

### 2.2 Study sample and data collection

The following inclusion criteria for the respondents must be met: (1) aged ≥ 18 years; (2) diagnosed with T2D; and (3) clearly aware and able to communicate normally. Patients with mental illness, critical condition, and cognitive impairment were excluded. Prior to the survey, the investigators were trained uniformly, the survey specifications were formulated and explained, and each item of the questionnaire was explained in detail. Trained investigators conducted surveys on patients who voluntarily participated and signed informed consent forms in local community hospitals and rural health centers. Face-to-face individual interviews were carried out, and each lasted for 20–25 min. At the end of the day, all questionnaires were checked by the investigators. The final sample size is 660 respondents. The investigators of this study were six master students from the Centre for Health Management and Policy Research of Shandong University. The investigators were provided with unified and systematic training before the investigation. A questionnaire survey was conducted on patients with T2D on a one-on-one questioning by trained investigators, which included questions on socio-demographic characteristics, disease-related characteristics, medical insurance-related characteristics, and My Opinion on Insulin (MOI). All answers to the questions were filled out by the investigators, and both questioning and filling out of the answers in the questionnaire were conducted simultaneously.

### 2.3 Measures and variables

MOI was used to measure the PIR in patients with T2D (31). The main table and auxiliary table together formed MOI. The main table was revised according to the translation of DAWN, and the auxiliary table was made according to the survey report on self-management status and its associated factors in Chinese patients with T2D (31–33). The main contents of MOI were the benefits and barriers to insulin use, including the advantages of insulin, cognitive disorders, life management disorders, attitude disorders, injection-related problems, adverse reaction disorders, and cost barriers (33). The main purpose of MOI was to understand the patient’s attitude and cognition toward insulin, so that investigators could understand the patient’s hesitation in insulin treatment. MOI had 20 five-point Likert scale questions with these options, namely, strongly agree, agree, moderate, disagree, and strongly disagree, and was successively assigned points of 1, 2, 3, 4, and 5. Items A1 to A4 were positive scoring, while items B1 to K2 were negative scoring. The total score of the scale ranges from 27 to 135, and the higher the score, the weaker the PIR. The Cronbach’s coefficient for positive items was 0.68, the coefficient for negative items was 0.90, and the coefficient for the entire scale was 0.89. A score of less than or equal to 3 on any item indicated the existence of a PIR (33).

Some covariates were included in the study. Socio-demographic variables included sex (male and female), age (<65, 65–74, and ≥75 years), marital status (married, divorced, or widowed), education level (nonliterate, with some primary schooling, and with some junior schooling or above), monthly income in Renminbi (RMB) (<2,000, 2,000–4,000, and ≥4,000), and financial burden (not serious and serious). Disease-related variables included the course of T2D (≤5, 6–10, and ≥11 years), self-reported health (healthy and unhealthy), and T2D level of discrimination (not serious, moderate, and serious). Medical insurance-related variables included categories of medical insurance: Urban and Rural Resident Basic Medical Insurance (URRBMI) and others.

### 2.4 Ethical considerations

The studies involving human participants were reviewed and approved by the institutional review board of Shandong University School of Public Health (Theory No. LL20221120). The patients/participants provided their written informed consent to participate in this study. The ‘personal data of the participants were kept anonymous and private for protection.

### 2.5 Statistical analysis

We used frequency (*N*), percentage (%), and mean [standard deviation (SD)] to show the socio-demographic and diabetes-related characteristics of the participants. Based on the chi-squared test, we compared the categorical factors associated with PIR in patients with T2D. Dummy variables were set for the categorical variables with more than two groups. In multivariate analysis, binary logistic regression was performed to further examine the factors associated with PIR. The odds ratio (OR), the 95% confidence interval (95% CI) of OR, and the *p*-value were reported. All statistical analyses were conducted using STATA 16.0. The significance level for statistics was set at two-sides *p* < 0.05.

## 3 Results

Among the 660 respondents with T2D, about two-thirds (66.8%) were women, and approximately 50% of them were aged 65 to 74 years old. The majority of respondents were married (82.7%). Nearly 80% of patients with T2D had a monthly income of less than 2,000 RMB. The majority of patients had a disease course of less than 5 years (40.8%), and only a minority had a disease course of 6–10 years (25.2%). Of the total respondents, 51.5% considered themselves unhealthy in the comprehensive health self-assessment. Nearly half of the respondents (50.5%) believed that patients with T2D were not seriously discriminated against, but some still hold (12.6%) that discrimination was relatively serious. The vast majority of respondents (94.7%) had URRBMI (**Table 1**).

**Table 1:** Basic characteristics of patients with type 2 diabetes in this study.

Characteristics	*n*	%
Sex		
Male	219	33.2
Female	441	66.8
Age		
<65	174	26.4
65–74	326	49.4
≥75	160	24.2
Marital status		
Married	546	82.7
Divorced or widowed	114	17.3
Educational level		
Nonliterate	230	34.9
With some primary schooling	226	34.2
With some junior schooling or above	204	30.9
Monthly income		
<2,000	525	79.6
2,000–4,000	84	12.7
≥4,000	51	7.7
Financial burden		
Not serious	506	76.7
Serious	154	23.3
Course of the disease (years)		
≤5	269	40.8
6–10	166	25.2
≥11	225	34.0
Self-rated health		
Healthy	320	48.5
Unhealthy	340	51.5
T2D level of discrimination		
Not serious	333	50.5
Moderate	244	37.0
Serious	83	12.5
Categories of medical insurance		
URRBMI	625	94.7
Others	35	5.3

URRBMI, the Urban and Rural Resident Basic Medical Insurance.

In this study, 82.1% of respondents reported having PIR. As shown in **Table 2**, 13.6% of patients had PIR in the dimension of insulin advantage; 54.9% of patients had cognitive disorders of insulin. Injection-related problems were the most severe among patients (58.6%); and 43.8% and 51.2% of patients had PIR in the dimensions of adverse reaction disorder and cost barriers, respectively.

**Table 2:** The scale of My Opinion on Insulin for psychological insulin resistance in all dimensions.

	PIR = NO	PIR = YES
Psychological insulin resistance	17.88	82.12
Advantages of insulin	86.36	13.64
Cognitive disorders	45.15	54.85
Life management disorders	54.09	45.91
Attitude disorders	42.27	57.73
Injection-related problems	41.36	58.64
Adverse reaction disorders	56.21	43.79
Cost barriers	48.79	51.21

PIR=NO, the constituent ratio of patients with type 2 diabetes without psychological insulin resistance. PIR=YES, the constituent ratio of patients with type 2 diabetes with psychological insulin resistance. Psychological insulin resistance, the overall condition of a patient’s psychological insulin resistance. Advantages of insulin, Cognitive disorders, Life management disorders, Attitude disorders, Injection related problems, Adverse reaction disorders, and Cost barriers are the seven dimensions of a patient’s psychological insulin resistance.

As shown in **Table 3**, PIR was more likely to be observed in female patients (85.5%) compared to male patients (75.3%). The prevalence of PIR varied greatly among patients with different disease courses (χ² = 17.538, *p* < 0.001). The prevalence of PIR was the highest in patients with T2D for 6–10 years (89.2%) and the lowest in patients with T2D for ≥11 years (73.8%). Patients who self-rated unhealthy (85.9%) had a higher prevalence of PIR than those (78.1%) who self-rated healthy (χ² = 6.757, *p* = 0.009). The prevalence of PIR varied with the level of T2D discrimination and was statistically significant (χ² = 14.152, *p* = 0.001). The prevalence of PIR was the highest in patients with a medium level of T2D discrimination (88.1%), and the lowest in patients with a non-serious level of T2D discrimination (76.6%).

**Table 3:** Psychological insulin resistance among patients with type 2 diabetes by characteristics.

Characteristics	PIR		χ²	*p*-value
	No	Yes		
Sex			10.258	0.001
Male	54 (24.7)	165 (75.3)		
Female	64 (14.5)	377 (85.5)		
Age			3.619	0.164
<65	29 (16.7)	145 (83.3)		
65–74	67 (20.6)	259 (79.4)		
≥75	22 (13.8)	138 (86.2)		
Marital status			0.011	0.918
Married	98 (17.9)	448 (82.1)		
Divorced or widowed	20 (17.5)	94 (82.5)		
Educational level			2.537	0.281
Nonliterate	35 (15.2)	195 (84.8)		
With some primary schooling	40 (17.7)	186 (82.3)		
With some junior schooling or above	43 (21.1)	161 (78.9)		
Monthly income			5.741	0.057
<2,000	86 (16.4)	439 (83.6)		
2,000–4,000	17 (20.2)	67 (79.8)		
≥4,000	15 (29.4)	36 (70.6)		
Financial burden			2.462	0.117
Not serious	97 (19.2)	409 (80.8)		
Serious	21 (13.6)	133 (86.4)		
Course of the disease (years)			17.538	0.000
≤5	41 (15.2)	228 (84.8)		
6–10	18 (10.8)	148 (89.2)		
≥11	59 (26.2)	166 (73.8)		
Self-rated health			6.757	0.009
Healthy	70 (21.9)	250 (78.1)		
Unhealthy	48 (14.1)	292 (85.9)		
Categories of medical insurance			4.622	0.032
URRBMI	107 (17.1)	518 (82.9)		
Others	11 (31.4)	24 (68.6)		
T2D level of discrimination			14.152	0.001
Not serious	78 (23.4)	255 (76.6)		
Medium	29 (11.9)	215 (88.1)		
Serious	11 (13.3)	72 (86.7)		

PIR, psychological insulin resistance. URRBMI, the Urban and Rural Resident Basic Medical Insurance.

**Table 4** shows the determinants of PIR in patients with T2D by binary logistic regression analysis. Female patients were more prone to PIR than male patients (OR = 1.770, 95% CI 1.063–2.950). Compared with patients with a monthly income of less than 2,000 RMB, patients with a monthly income of more than or equal to 4,000 RMB were less likely to develop PIR (OR = 0.444, 95% CI 0.216–0.915). Patients whose course of disease was more than or equal to 11 years were less likely to develop PIR than those whose course of disease was less than or equal to 5 years (OR = 0.387, 95% CI 0.238–0.630). Patients who rated themselves as unhealthy were more prone to PIR (OR = 1.706, 95% CI 1.092–2.664). Compared with patients who were not severely discriminated against, patients who were moderately discriminated against were more likely to develop PIR (OR = 1.924, 95% CI 1.166–3.175).

**Table 4:** Factors associated with psychological insulin resistance among patients with type 2 diabetes.

Characteristics	Reference	OR	95% CI	*p*
Sex				
Female	Male	1.770	1.063–2.950	0.028
Age				
65–75	<65	1.305	0.759–2.245	0.335
≥75		1.346	0.681–2.659	0.392
Marital status				
Divorced or widowed	Married	0.937	0.519–1.691	0.829
Educational level				
With some primary schooling	Nonliterate	1.015	0.568–1.815	0.959
With some primary schooling		0.996	0.527–1.882	0.989
Monthly income				
2,000–4,000	<2,000	1.049	0.554–1.985	0.883
≥4,000		0.444	0.216–0.915	0.028
Financial burden				
Serious	Not serious	1.264	0.714–2.238	0.422
Course of the disease (years)				
6–10	≤5	1.343	0.726–2.484	0.347
≥11		0.387	0.238–0.630	0.000
Self-rated health				
Unhealthy	Healthy	1.706	1.092–2.664	0.019
Categories of medical insurance				
URRBMI	Others	1.831	0.794–4.219	0.156
T2D level of discrimination				
Medium	Not serious	1.924	1.166–3.175	0.010
Serious		1.761	0.856–3.625	0.124
Constant		0.253	0.027–2.342	0.226

URRBMI, the Urban and Rural Resident Basic Medical Insurance.

## 4 Discussion

This study investigated the prevalence and factors associated with PIR in patients with T2D in China. We found that patients with T2D in China currently had serious PIR, and the prevalence of PIR was 82.1%. Compared to previous studies, the prevalence of PIR in patients with T2D was lower, which may have been related to an increase in awareness of T2D (4, 34). Healthcare providers’ health education plays a significant role in changing patients’ attitudes toward insulin, providing a supportive environment to help promote insulin initiation (24, 35, 36). The high prevalence of PIR reveals that it has become a significant public health issue. Therefore, additional measures are warranted to address PIR.

In this study, sex and income were found to be associated with PIR. We found that women with T2D were more likely to have PIR than men, which is aligned with previous studies indicating that sex was likely to be associated with refusal of insulin treatment (36, 37). Patients with T2D with different incomes were likely to have a different prevalence of PIR. We found that the prevalence of PIR in patients with T2D with a high monthly income was lower than that in patients with a low monthly income. It might take a long time before patients are able to control their own blood sugar through insulin, and this also puts forward a high requirement for the improvement of the patients’ economic situation. Interestingly, the economic burden did not show any correlation with PIR in this study. Previous studies found that the economic affordability of patients with T2D affected insulin treatment (20, 21). Insulin therapy was viewed as the most burdensome treatment modality among people with T2D (38). Of the total number of patients, 77% relied on their families to meet their economic and physical needs, with financial constraints being the most common cause (74.3%) (37).

The course of the disease and the patients’ self-rated health may influence PIR from a cognitive perspective. We found that patients with a long duration of T2D were less likely to develop PIR than those with a short duration. After many years of treatment, patients with a long course of disease were likely to have a more comprehensive understanding of T2D and insulin and were not prone to PIR. A study found that patients could adapt to the impact of diabetes based on time and experience after a long diabetes duration (39). Self-rated health was the understanding of patients regarding their own health. We found that patients who rated themselves unhealthy were more likely to have PIR than those who rated themselves healthy. It was associated with the patients’ wrong understanding of T2D and insulin therapy (40). Patients were likely to believe that receiving insulin treatment indicated the progression of the condition to a more severe stage (20, 23). For example, in a study conducted in Australia, half of the patients with T2D receiving insulin treatment believed that using insulin meant the deterioration of their condition (38). A similar observation was noted in a prospective study conducted in Korea, where nearly one-third of the patients felt that their disease is in the last stage if they received insulin treatment (41). Previous studies found that patients believed that receiving insulin treatment was an impossible choice (22, 39).

Social stigma is a significant aspect that impacts patients’ PIR (19). Discrimination is a very specific form of social stigma, which has been largely hidden from view (20). The degree of discrimination against patients with T2D was likely to be associated with PIR. In this study, we found that compared with patients who were not severely discriminated against, patients who were moderately discriminated against were more likely to have PIR. Interestingly, we found that the degree of discrimination against patients with T2D was not serious, and nearly 90% of patients thought that the degree of discrimination was not serious or average. Since this was the degree of self-reported discrimination, it was possible that the degree of self-perceived discrimination of patients with T2D could bring psychological stress to themselves; thus, they were unwilling to undergo, if not delay, insulin treatment. Several DAWN2 transnational studies have also found that diabetes brought psychological burden to patients with diabetes, which were likely to be one of the reasons that hinder insulin treatment (6, 39, 42, 43). The discrimination against this disease in society was likely to be due to the non-obvious results of relevant science popularization actions, and the awareness and acceptance of this disease in society need to be improved (42, 43).

This study is subject to three limitations. First, it is a cross-sectional study; causal relationships are difficult to determine. In the future, longitudinal data will be warranted for the causal inference of PIR. Second, the research mainly considers the associated factors from the demand side (i.e., patient side), without considering the supply side factors associated with insulin use, such as a healthcare professional side. Third, the research conclusions were drawn from a survey in Shandong Province, China, which may limit worldwide generalizability.

## 5 Conclusions

The prevalence of PIR among Chinese patients with T2D is relatively high. PIR is associated with sex, monthly income, course of diabetes, self-rated health, and the level of discrimination against diabetes. Measures should be taken to reduce the prevalence of PIR and facilitate the development of effective glycemic control management strategies for patients with T2D.

## Data availability statement

The raw data supporting the conclusions of this article will be made available by the authors, without undue reservation.

## Ethics statement

The studies involving humans were approved by Shandong University School of Public Health (Theory No.: LL20221120). The studies were conducted in accordance with the local legislation and institutional requirements. The participants provided their written informed consent to participate in this study.

## Author contributions

SZ: Writing – original draft, Writing – review & editing. XZ: Investigation, Writing – review & editing. HW: Methodology, Project administration, Writing – review & editing. WF: Investigation, Writing – review & editing. XM: Investigation, Writing – review & editing. SS: Writing – review & editing. BZ: Writing – review & editing.
